# Supplementary material for: A fault-intrusion-tolerant system and deadline-aware algorithm for scheduling scientific workflow in the cloud
Source: PeerJ Comput Sci. 2021 Nov 2;7:e747. doi: 10.7717/peerj-cs.747 (PMC8576554; doi:10.7717/peerj-cs.747)
Supplement: Supplemental Information 6 [file peerj-cs-07-747-s006.zip › Myproject1.1/docs/org/cloudbus/cloudsim/Host.html]

Host


JavaScript is disabled on your browser.


- Overview
- Package
- Class
- Use
- Tree
- Deprecated
- Index
- Help

- Prev Class
- Next Class

- Frames
- No Frames

- All Classes

- Summary:
- Nested |
- Field |
- Constr |
- Method

- Detail:
- Field |
- Constr |
- Method


org.cloudbus.cloudsim

## Class Host

- java.lang.Object
- - org.cloudbus.cloudsim.Host

- Direct Known Subclasses:
  :   HostDynamicWorkload, NetworkHost

  ---

    

  ```
  public class Host
  extends java.lang.Object
  ```

  Host executes actions related to management of virtual machines (e.g., creation and destruction).
  A host has a defined policy for provisioning memory and bw, as well as an allocation policy for
  Pe's to virtual machines. A host is associated to a datacenter. It can host virtual machines.

  Since:
  :   CloudSim Toolkit 1.0

- - ### Constructor Summary

    Constructors

    Constructor and Description

    `Host(int id,
    RamProvisioner ramProvisioner,
    BwProvisioner bwProvisioner,
    long storage,
    java.util.List<? extends Pe> peList,
    VmScheduler vmScheduler)`

    Instantiates a new host.
  - ### Method Summary

    Methods

    | Modifier and Type | Method and Description |
    | --- | --- |
    | `void` | `addMigratingInVm(Vm vm)` Adds the migrating in vm. |
    | `boolean` | `allocatePesForVm(Vm vm, java.util.List<java.lang.Double> mipsShare)` Allocates PEs for a VM. |
    | `void` | `deallocatePesForVm(Vm vm)` Releases PEs allocated to a VM. |
    | `java.util.List<java.lang.Double>` | `getAllocatedMipsForVm(Vm vm)` Returns the MIPS share of each Pe that is allocated to a given VM. |
    | `double` | `getAvailableMips()` Gets the free mips. |
    | `long` | `getBw()` Gets the machine bw. |
    | `BwProvisioner` | `getBwProvisioner()` Gets the bw provisioner. |
    | `Datacenter` | `getDatacenter()` Gets the data center. |
    | `int` | `getId()` Gets the id. |
    | `double` | `getMaxAvailableMips()` Returns maximum available MIPS among all the PEs. |
    | `int` | `getNumberOfFreePes()` Gets the free pes number. |
    | `int` | `getNumberOfPes()` Gets the pes number. |
    | `<T extends Pe>  java.util.List<T>` | `getPeList()` Gets the pe list. |
    | `int` | `getRam()` Gets the machine memory. |
    | `RamProvisioner` | `getRamProvisioner()` Gets the ram provisioner. |
    | `long` | `getStorage()` Gets the machine storage. |
    | `double` | `getTotalAllocatedMipsForVm(Vm vm)` Gets the total allocated MIPS for a VM over all the PEs. |
    | `int` | `getTotalMips()` Gets the total mips. |
    | `Vm` | `getVm(int vmId, int userId)` Returns a VM object. |
    | `<T extends Vm>  java.util.List<T>` | `getVmList()` Gets the vm list. |
    | `VmScheduler` | `getVmScheduler()` Gets the VM scheduler. |
    | `java.util.List<Vm>` | `getVmsMigratingIn()` Gets the vms migrating in. |
    | `boolean` | `isFailed()` Checks if is failed. |
    | `boolean` | `isSuitableForVm(Vm vm)` Checks if is suitable for vm. |
    | `void` | `reallocateMigratingInVms()` Reallocate migrating in vms. |
    | `void` | `removeMigratingInVm(Vm vm)` Removes the migrating in vm. |
    | `protected void` | `setBwProvisioner(BwProvisioner bwProvisioner)` Sets the bw provisioner. |
    | `void` | `setDatacenter(Datacenter datacenter)` Sets the data center. |
    | `boolean` | `setFailed(boolean failed)` Sets the PEs of this machine to a FAILED status. |
    | `boolean` | `setFailed(java.lang.String resName, boolean failed)` Sets the PEs of this machine to a FAILED status. |
    | `protected void` | `setId(int id)` Sets the id. |
    | `protected <T extends Pe>  void` | `setPeList(java.util.List<T> peList)` Sets the pe list. |
    | `boolean` | `setPeStatus(int peId, int status)` Sets the particular Pe status on this Machine. |
    | `protected void` | `setRamProvisioner(RamProvisioner ramProvisioner)` Sets the ram provisioner. |
    | `protected void` | `setStorage(long storage)` Sets the storage. |
    | `protected void` | `setVmScheduler(VmScheduler vmScheduler)` Sets the VM scheduler. |
    | `double` | `updateVmsProcessing(double currentTime)` Requests updating of processing of cloudlets in the VMs running in this host. |
    | `boolean` | `vmCreate(Vm vm)` Allocates PEs and memory to a new VM in the Host. |
    | `protected void` | `vmDeallocate(Vm vm)` Deallocate all hostList for the VM. |
    | `protected void` | `vmDeallocateAll()` Deallocate all hostList for the VM. |
    | `void` | `vmDestroy(Vm vm)` Destroys a VM running in the host. |
    | `void` | `vmDestroyAll()` Destroys all VMs running in the host. |

    - ### Methods inherited from class java.lang.Object

      `clone, equals, finalize, getClass, hashCode, notify, notifyAll, toString, wait, wait, wait`

- - ### Constructor Detail


    - #### Host

      ```
      public Host(int id,
          RamProvisioner ramProvisioner,
          BwProvisioner bwProvisioner,
          long storage,
          java.util.List<? extends Pe> peList,
          VmScheduler vmScheduler)
      ```

      Instantiates a new host.

      Parameters:
      :   `id` - the id
      :   `ramProvisioner` - the ram provisioner
      :   `bwProvisioner` - the bw provisioner
      :   `storage` - the storage
      :   `peList` - the pe list
      :   `vmScheduler` - the vm scheduler
  - ### Method Detail


    - #### updateVmsProcessing

      ```
      public double updateVmsProcessing(double currentTime)
      ```

      Requests updating of processing of cloudlets in the VMs running in this host.

      Parameters:
      :   `currentTime` - the current time

      Returns:
      :   expected time of completion of the next cloudlet in all VMs in this host.
          Double.MAX\_VALUE if there is no future events expected in this host


    - #### addMigratingInVm

      ```
      public void addMigratingInVm(Vm vm)
      ```

      Adds the migrating in vm.

      Parameters:
      :   `vm` - the vm


    - #### removeMigratingInVm

      ```
      public void removeMigratingInVm(Vm vm)
      ```

      Removes the migrating in vm.

      Parameters:
      :   `vm` - the vm


    - #### reallocateMigratingInVms

      ```
      public void reallocateMigratingInVms()
      ```

      Reallocate migrating in vms.


    - #### isSuitableForVm

      ```
      public boolean isSuitableForVm(Vm vm)
      ```

      Checks if is suitable for vm.

      Parameters:
      :   `vm` - the vm

      Returns:
      :   true, if is suitable for vm


    - #### vmCreate

      ```
      public boolean vmCreate(Vm vm)
      ```

      Allocates PEs and memory to a new VM in the Host.

      Parameters:
      :   `vm` - Vm being started

      Returns:
      :   $true if the VM could be started in the host; $false otherwise


    - #### vmDestroy

      ```
      public void vmDestroy(Vm vm)
      ```

      Destroys a VM running in the host.

      Parameters:
      :   `vm` - the VM


    - #### vmDestroyAll

      ```
      public void vmDestroyAll()
      ```

      Destroys all VMs running in the host.


    - #### vmDeallocate

      ```
      protected void vmDeallocate(Vm vm)
      ```

      Deallocate all hostList for the VM.

      Parameters:
      :   `vm` - the VM


    - #### vmDeallocateAll

      ```
      protected void vmDeallocateAll()
      ```

      Deallocate all hostList for the VM.


    - #### getVm

      ```
      public Vm getVm(int vmId,
             int userId)
      ```

      Returns a VM object.

      Parameters:
      :   `vmId` - the vm id
      :   `userId` - ID of VM's owner

      Returns:
      :   the virtual machine object, $null if not found


    - #### getNumberOfPes

      ```
      public int getNumberOfPes()
      ```

      Gets the pes number.

      Returns:
      :   the pes number


    - #### getNumberOfFreePes

      ```
      public int getNumberOfFreePes()
      ```

      Gets the free pes number.

      Returns:
      :   the free pes number


    - #### getTotalMips

      ```
      public int getTotalMips()
      ```

      Gets the total mips.

      Returns:
      :   the total mips


    - #### allocatePesForVm

      ```
      public boolean allocatePesForVm(Vm vm,
                             java.util.List<java.lang.Double> mipsShare)
      ```

      Allocates PEs for a VM.

      Parameters:
      :   `vm` - the vm
      :   `mipsShare` - the mips share

      Returns:
      :   $true if this policy allows a new VM in the host, $false otherwise


    - #### deallocatePesForVm

      ```
      public void deallocatePesForVm(Vm vm)
      ```

      Releases PEs allocated to a VM.

      Parameters:
      :   `vm` - the vm


    - #### getAllocatedMipsForVm

      ```
      public java.util.List<java.lang.Double> getAllocatedMipsForVm(Vm vm)
      ```

      Returns the MIPS share of each Pe that is allocated to a given VM.

      Parameters:
      :   `vm` - the vm

      Returns:
      :   an array containing the amount of MIPS of each pe that is available to the VM


    - #### getTotalAllocatedMipsForVm

      ```
      public double getTotalAllocatedMipsForVm(Vm vm)
      ```

      Gets the total allocated MIPS for a VM over all the PEs.

      Parameters:
      :   `vm` - the vm

      Returns:
      :   the allocated mips for vm


    - #### getMaxAvailableMips

      ```
      public double getMaxAvailableMips()
      ```

      Returns maximum available MIPS among all the PEs.

      Returns:
      :   max mips


    - #### getAvailableMips

      ```
      public double getAvailableMips()
      ```

      Gets the free mips.

      Returns:
      :   the free mips


    - #### getBw

      ```
      public long getBw()
      ```

      Gets the machine bw.

      Returns:
      :   the machine bw


    - #### getRam

      ```
      public int getRam()
      ```

      Gets the machine memory.

      Returns:
      :   the machine memory


    - #### getStorage

      ```
      public long getStorage()
      ```

      Gets the machine storage.

      Returns:
      :   the machine storage


    - #### getId

      ```
      public int getId()
      ```

      Gets the id.

      Returns:
      :   the id


    - #### setId

      ```
      protected void setId(int id)
      ```

      Sets the id.

      Parameters:
      :   `id` - the new id


    - #### getRamProvisioner

      ```
      public RamProvisioner getRamProvisioner()
      ```

      Gets the ram provisioner.

      Returns:
      :   the ram provisioner


    - #### setRamProvisioner

      ```
      protected void setRamProvisioner(RamProvisioner ramProvisioner)
      ```

      Sets the ram provisioner.

      Parameters:
      :   `ramProvisioner` - the new ram provisioner


    - #### getBwProvisioner

      ```
      public BwProvisioner getBwProvisioner()
      ```

      Gets the bw provisioner.

      Returns:
      :   the bw provisioner


    - #### setBwProvisioner

      ```
      protected void setBwProvisioner(BwProvisioner bwProvisioner)
      ```

      Sets the bw provisioner.

      Parameters:
      :   `bwProvisioner` - the new bw provisioner


    - #### getVmScheduler

      ```
      public VmScheduler getVmScheduler()
      ```

      Gets the VM scheduler.

      Returns:
      :   the VM scheduler


    - #### setVmScheduler

      ```
      protected void setVmScheduler(VmScheduler vmScheduler)
      ```

      Sets the VM scheduler.

      Parameters:
      :   `vmScheduler` - the vm scheduler


    - #### getPeList

      ```
      public <T extends Pe> java.util.List<T> getPeList()
      ```

      Gets the pe list.

      Type Parameters:
      :   `T` - the generic type

      Returns:
      :   the pe list


    - #### setPeList

      ```
      protected <T extends Pe> void setPeList(java.util.List<T> peList)
      ```

      Sets the pe list.

      Type Parameters:
      :   `T` - the generic type

      Parameters:
      :   `peList` - the new pe list


    - #### getVmList

      ```
      public <T extends Vm> java.util.List<T> getVmList()
      ```

      Gets the vm list.

      Type Parameters:
      :   `T` - the generic type

      Returns:
      :   the vm list


    - #### setStorage

      ```
      protected void setStorage(long storage)
      ```

      Sets the storage.

      Parameters:
      :   `storage` - the new storage


    - #### isFailed

      ```
      public boolean isFailed()
      ```

      Checks if is failed.

      Returns:
      :   true, if is failed


    - #### setFailed

      ```
      public boolean setFailed(java.lang.String resName,
                      boolean failed)
      ```

      Sets the PEs of this machine to a FAILED status. NOTE: resName is used for debugging
      purposes, which is **ON** by default. Use `setFailed(boolean)` if you do not want
      this information.

      Parameters:
      :   `resName` - the name of the resource
      :   `failed` - the failed

      Returns:
      :   true if successful, false otherwise


    - #### setFailed

      ```
      public boolean setFailed(boolean failed)
      ```

      Sets the PEs of this machine to a FAILED status.

      Parameters:
      :   `failed` - the failed

      Returns:
      :   true if successful, false otherwise


    - #### setPeStatus

      ```
      public boolean setPeStatus(int peId,
                        int status)
      ```

      Sets the particular Pe status on this Machine.

      Parameters:
      :   `peId` - the pe id
      :   `status` - Pe status, either Pe.FREE or Pe.BUSY

      Returns:
      :   true if the Pe status has changed, false otherwise (Pe id might not
          be exist)


    - #### getVmsMigratingIn

      ```
      public java.util.List<Vm> getVmsMigratingIn()
      ```

      Gets the vms migrating in.

      Returns:
      :   the vms migrating in


    - #### getDatacenter

      ```
      public Datacenter getDatacenter()
      ```

      Gets the data center.

      Returns:
      :   the data center where the host runs


    - #### setDatacenter

      ```
      public void setDatacenter(Datacenter datacenter)
      ```

      Sets the data center.

      Parameters:
      :   `datacenter` - the data center from this host


- Overview
- Package
- Class
- Use
- Tree
- Deprecated
- Index
- Help

- Prev Class
- Next Class

- Frames
- No Frames

- All Classes

- Summary:
- Nested |
- Field |
- Constr |
- Method

- Detail:
- Field |
- Constr |
- Method
